# Combined Pharmacological and Pneumatic Displacement Therapy for Sub-Macular Haemorrhage Secondary to Age-Related Macular Degeneration: A Case Series and Review of the Literature

**DOI:** 10.3390/life16010003

**Published:** 2025-12-19

**Authors:** Agnieszka Kudasiewicz-Kardaszewska, Małgorzata Anna Ozimek, Tomasz Urbański, Kinga Jamontt, Aleksander Tkaczenko, Karolina Bonińska, Sławomir Cisiecki

**Affiliations:** 1 OCHO Medical Group, Zagorski Eye Surgery Centre in Nowy Sącz, 33-300 Nowy Sącz, Poland; 2 Department of Opththalmology, Miejskie Centrum Medyczne im. Karola Jonschera, 93-113 Łódź, Poland; 3 OCHO Medical Group, Zagorski Eye Surgery Centres, Solskiego 7C, 31-216 Kraków, Poland

**Keywords:** sub-macular haemorrhage, neovascular age-related macular degeneration (nAMD), tissue plasminogen activator (tPA; alteplase), expansile gas, anti-VEGF, aflibercept

## Abstract

Purpose: We aimed to present the clinical outcomes of a combined pharmacological and gas-assisted treatment for sub-macular haemorrhage secondary to neovascular age-related macular degeneration (nAMD). Methods: This retrospective case series included ten eyes with sub-macular haemorrhage (SMH) treated between January 2024 and September 2025. All patients received intravitreal alteplase (100 µg) and C_3_F_8_ gas, followed by aflibercept (2 mg or 8 mg) within a treat-and-extend regimen. BCVA- and OCT-based anatomical changes were recorded at baseline, 7–14 days, 1 month, 3 months, and 6 months. BCVA changes were analysed using repeated-measures testing. Results: Mean BCVA improved from 0.99 ± 0.21 logMAR at baseline to 0.89 ± 0.20 at 7–14 days, 0.80 ± 0.20 at 1 month, 0.60 ± 0.18 at 3 months, and 0.53 ± 0.20 at 6 months (*p* < 0.05 for overall change). Eight eyes (80 percent) showed restoration of foveal contour, while two developed foveal atrophy. No major adverse events occurred. Conclusion: Combined intravitreal alteplase and C_3_F_8_, followed by aflibercept, may provide favourable short-term visual and anatomical improvement in SMH secondary to nAMD. Early intervention appears beneficial, but larger controlled studies are needed to confirm these findings.

## 1. Introduction

**Figure 1:**
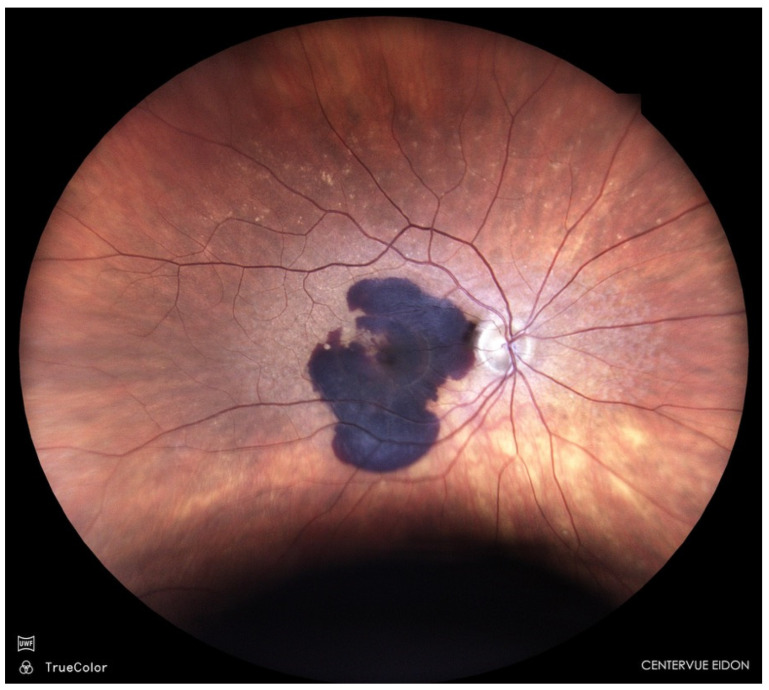
Sub-macular haemorrhage in nAMD.

Sub-macular haemorrhage (SMH) is a vision-threatening complication of neovascular age-related macular degeneration (nAMD) (Figure 1); polypoidal choroidal vasculopathy (PCV), and rupture of macular macro- or micro-aneurysms [1,2].

The accumulation of blood in the subretinal space exerts toxic and mechanical effects on the photoreceptors and retinal pigment epithelium, often leading to irreversible vision loss if left untreated [3,4,5]. The presence of iron, haemosiderin, and fibrin within the subretinal space triggers photoreceptor apoptosis within hours, emphasising the need for early displacement of the clot to restore the foveal architecture and prevent permanent macular damage [6].

Current therapeutic options include enzymatic clot liquefaction with tissue plasminogen activator (tPA; alteplase) with or without pneumatic displacement using expansile gas (e.g., sulphur hexafluoride [SF_6_] or perfluoropropane [C_3_F_8_]), anti-VEGF therapy alone, and surgical interventions such as pars plana vitrectomy with subretinal injection of tPA [7,8,9,10,11]. While surgery can be effective, it carries higher risks and costs and may not be accessible in all clinical settings [8,10].

A combined pharmacological and pneumatic approach allows both enzymatic degradation of the subretinal clot and mechanical displacement of blood from the foveal region. This strategy can be performed in an outpatient setting and offers a favourable balance between efficacy, safety, and accessibility—particularly for elderly patients who, owing to comorbidities such as cardiovascular disease, hypertension, or diabetes, are often unfit for surgery [11]. Although several case series have explored pneumatic displacement techniques, most published studies focus on vitrectomy-based approaches or use heterogeneous treatment combinations [8,9,12,13,14,15]. Evidence on a simple, minimally invasive protocol combining intravitreal alteplase, pure C_3_F_8_ gas, and subsequent anti-VEGF therapy remains limited, particularly regarding short-term anatomical recovery documented with modern OCT imaging. This study adds current, real-world data on this approach using both available aflibercept formulations within a consistent treatment pathway.

### Aim

The aim is to evaluate the anatomical and functional outcomes achieved with the combined minimally invasive management strategy (intravitreal tPA + gas C_3_F_8_, followed by aflibercept with a treat-and-extend (T&Ex) regimen) in patients with sub-macular haemorrhage secondary to nAMD over a six-month follow-up.

## 2. Materials and Methods

### 2.1. Study Design and Participants

This retrospective case series included ten patients (10 eyes) treated from January 2024 to September 2025 at the Prof. Zagórski Eye Surgery Centre, Nowy Sącz, Poland. All patients presented with a diagnosis of SMH secondary to exudative AMD.

Inclusion criteria were as follows: a diagnosis of neovascular AMD with SMH confirmed by OCT and fundus imaging, and symptom onset within 60 days prior to treatment. The 60-day upper limit was chosen because haemorrhages older than two months typically evolve into organised clots with limited potential for displacement and a high likelihood of fibrosis. This criterion aligns with previous clinical studies that use a similar timeframe to distinguish acute from chronic SMH.

Exclusion criteria consisted of haemorrhage lasting longer than two months or the presence of scarring.

Data were extracted from electronic medical records. Symptom onset was verified based on patient self-report documented at the first visit, cross-checked with ophthalmic findings consistent with fresh haemorrhage. All BCVA and OCT assessments were performed by two experienced retina specialists; disagreements were resolved by consensus. All ten eyes completed the six-month examination schedule.

All procedures were conducted in accordance with institutional guidelines and the Declaration of Helsinki. Formal ethics approval was waived due to the retrospective nature of the study. Informed consent was obtained from all participants.

### 2.2. Treatment Protocol

All patients underwent intravitreal injection of tPA (Alteplase, Actilyse^®^ 100 µg/0.1 mL, Boehringer Ingelheim International, Vienna, Austria) combined with pure perfluoropropane gas (C_3_F_8_) 0.3 mL at 100% (BVI, Kraków, Poland) concentration, followed by anterior chamber paracentesis to prevent post-procedural intraocular pressure (IOP) elevation. The dose of 0.3 mL of pure 100 percent C_3_F_8_ was selected based on published evidence demonstrating that small volume expansile gas provides adequate tamponade for pneumatic displacement while reducing the risk of excessive intraocular pressure elevation [11,16,17]. The procedure was performed under aseptic conditions in the operating theatre. After treatment, patients were instructed to maintain a prone position for at least three days to facilitate displacement of sub-macular blood.

Between 7 and 14 days after the initial tPA + gas injection, all patients began anti-VEGF therapy with either aflibercept 2.0 mg/0.1 mL (Eylea 2 mg, Bayer) or aflibercept 8 mg/0.1 mL (Eylea 8 mg, Bayer AG, Leverkusen, Germany) using a treat-and-extend (T&Ex) regimen. The loading phase for both formulations consisted of three-monthly injections, followed by extension according to the T&Ex protocol [18].

### 2.3. Outcome Measures

Patients underwent comprehensive ophthalmic evaluation at baseline and at 7 days or 14 days, 1 month, 3 months, and 6 months after initial tPA and gas injection. Assessments included best-corrected visual acuity (BCVA) measured using an ETDRS chart, fundus photography, and macular spectral-domain optical coherence tomography (SD-OCT) at each time point.

### 2.4. Statistical Analysis

BCVA values were analysed using repeated-measures tests to compare changes over time (Friedman ANOVA for non-parametric data). Results are presented as mean ± standard deviation and 95 percent confidence intervals where appropriate. A significance level of *p* < 0.05 was applied. All analyses pooled eyes treated with aflibercept 2 mg and 8 mg, as both formulations share the same mechanism of action and were administered according to the same treat-and-extend protocol.

## 3. Results

Ten patients (ten eyes) with sub-macular haemorrhage secondary to neovascular AMD were included. The mean age was 76.0 ± 6.1 years (range: 60–90 years). Five patients were female and five were male. All eyes presented with fovea-involving haemorrhage. The mean duration from symptom onset to treatment was 25 ± 18 days (range: 7–60 days). To explore the effect of treatment timing, patients were grouped by symptom duration: early (≤14 days, *n* = 6) and delayed (>14 days, *n* = 4). Early treated eyes showed greater BCVA gain at six months (mean improvement 0.55 logMAR vs. 0.32 logMAR), although the small sample size prevents formal statistical interpretation.

Following the peer review suggestions, we also performed a second exploratory comparison between early treatment (≤14 days, *n* = 6), intermediate treatment (15–30 days, *n* = 1), and late treatment (>30 days, *n* = 3). Eyes treated within 14 days demonstrated the greatest BCVA improvement at six months (0.55 logMAR). The intermediate case showed moderate gain (0.40 logMAR), while the two late cases, i.e., case 1 and case 9 (>30 days), demonstrated limited improvement (0.18 and 0.22 logMAR), consistent with their development of atrophy. Again, the small sample size prevents formal statistical interpretation.

We explored whether sex or age influenced outcomes. No meaningful difference in BCVA improvement was observed between males and females (mean gains 0.48 vs. 0.44 logMAR). Similarly, patients aged ≥ 80 years (*n* = 3) showed comparable improvement to those under 70 (*n* = 2), although numbers were too small for statistical comparison. These exploratory observations suggest that timing of treatment, rather than demographic variables, played a larger role in functional outcome. Table 1 summarises the demographic data, diagnosis, and symptom onset.

**Figure 2:**
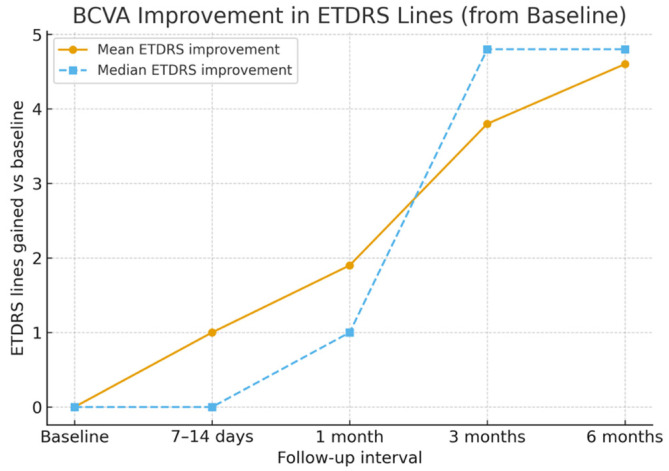
Mean and median EDTRS lines gain with time.

The mean baseline BCVA was 0.99 ± 0.21 logMAR (Snellen equivalent ≈ 20/180). By 7–14 days, the mean BCVA improved to 0.89 ± 0.20 logMAR (≈20/155), representing an early gain of approximately one ETDRS line. At 1 month, the mean BCVA improved further to 0.80 ± 0.20 logMAR (≈20/110), corresponding to an average gain of two ETDRS lines. At 3 months, the mean BCVA reached 0.60 ± 0.18 logMAR (≈20/80), and by 6 months, it improved to 0.53 ± 0.20 logMAR (≈20/70); *p* < 0.05 for overall change, with six eyes (60%) gaining four or more ETDRS lines and none lost vision. Mean and median ETDRS line gains are shown in Figure 2, and BCVA changes over time are detailed in Table 2.

Complications occurred in a small number of patients. Two eyes developed cataract progression during follow-up (Cases 7 and 8), one eye experienced transient intraocular pressure elevation that resolved promptly with paracentesis (Case 9), and two eyes showed macular neovascularization (MNV) scarring (Cases 3 and 10). No cases of endophthalmitis, retinal detachment, retinal pigment epithelium tear, or recurrent or breakthrough haemorrhage were observed.

**Figure 3:**
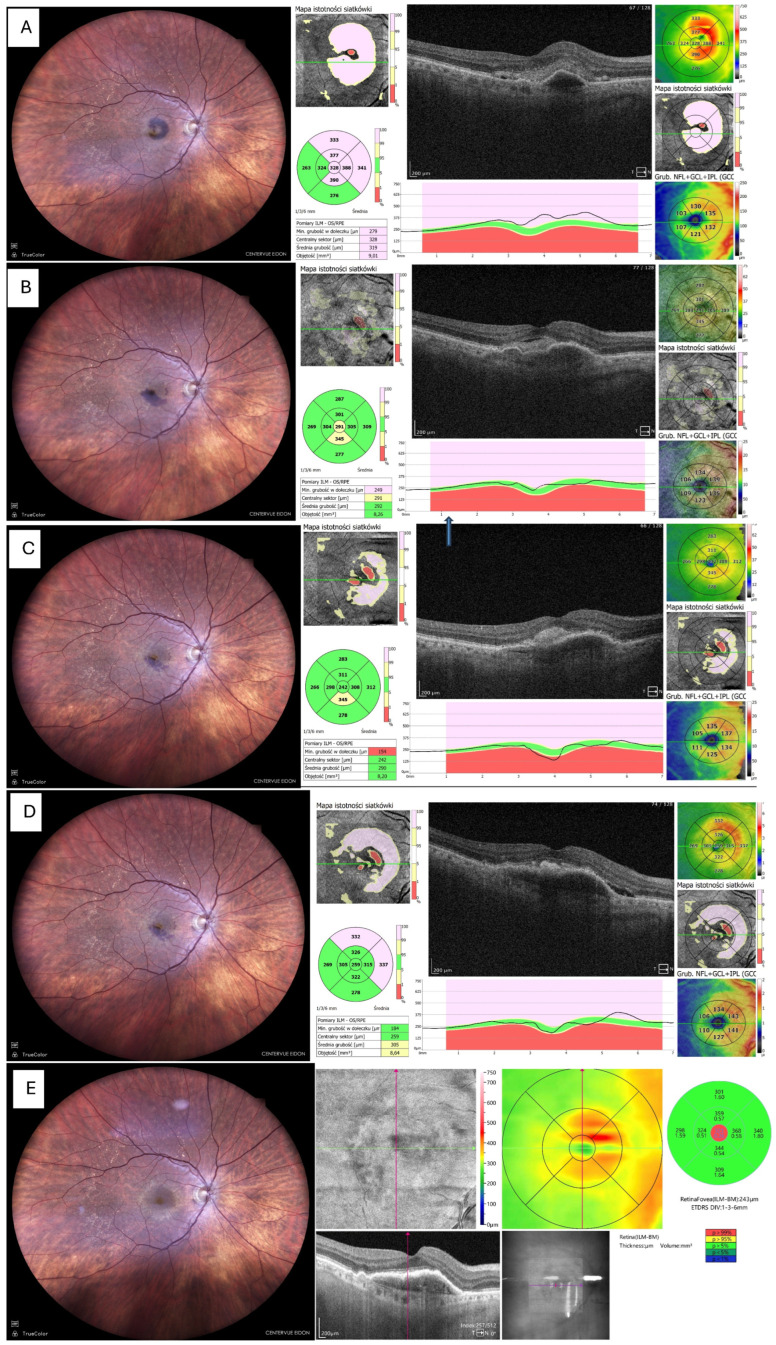
Resolution of haemorrhage with restoration of foveal contour. (**A**) Fundus photo and OCT before tPA + gas administration. (**B**) Fundus photo and OCT 14 days after tPA + gas, showing significant reduction in haemorrhage and retinal thickness; the patient received the first intravitreal aflibercept 2 mg (Eylea 2 mg, Bayer, Leverkusen, Germany). (**C**) Fundus photo and OCT 1 month postoperatively (only small remnants of blood visible). (**D**) Fundus photo and OCT 3 months postoperatively, patient on aflibercept 2 mg T&Ex regimen. (**E**) Fundus photo and OCT 6 months postoperatively, no blood visible but MNV still active, requiring continuation of anti-VEGF therapy. Foveal contour preserved.

**Figure 4:**
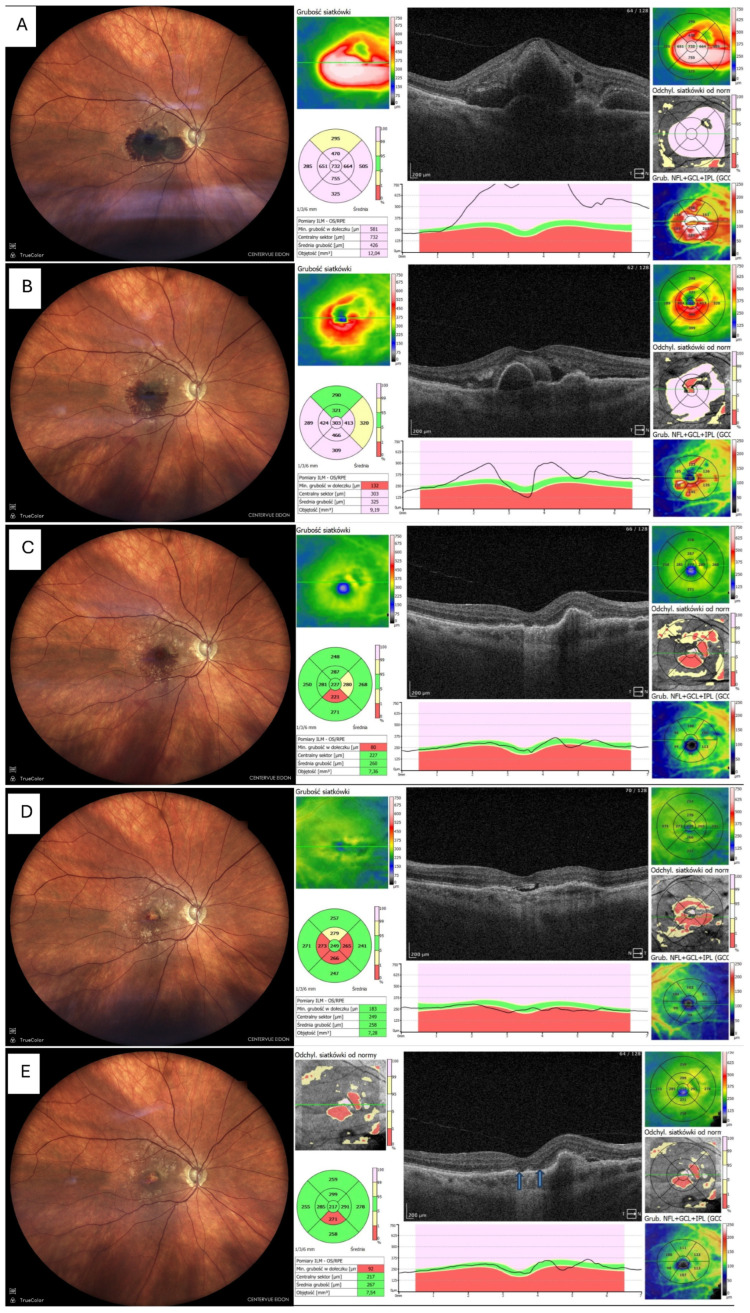
Resolution of haemorrhage with ellipsoid zone disruption. MNV = macular neovascularisation, OCT = optical coherent tomography, T&Ex = treat and extend (**A**) Fundus photo and OCT before tPA + gas, showing thick sub-macular haemorrhage involving the fovea (**B**) Fundus photo and OCT 7 days after tPA + gas, showing marked reduction in haemorrhage and retinal thickness; patient received first intravitreal aflibercept 8 mg (Eylea 8 mg, Bayer, Leverkusen, Germany) (**C**) Fundus photo and OCT 1 month postoperatively (thin blood remnants visible) (**D**) Fundus photo and OCT 3 months postoperatively, showing foveal atrophy in both fundus image and OCT; patient on aflibercept 8 mg T&Ex regimen (**E**) Fundus photo and OCT 6 months postoperatively, no blood visible, with central atrophy and persistent MNV activity requiring ongoing anti-VEGF therapy. Elipsoid zone disruption is indicated by arrows.

Fundus photography and OCT imaging confirmed near-complete displacement of the haemorrhage by day 30 and restoration of the foveal contour in eight eyes (80%), as depicted in Figure 3, whereas two eyes (20%) showed residual ellipsoid-zone disruption and subsequent fibrotic scarring and atrophy, which is shown in Figure 4.

Representative images were selected based on clarity and the ability to illustrate typical and atypical anatomical outcomes. Figure 3 demonstrates a common recovery pattern with restoration of foveal contour, whereas Figure 4 highlights an uncommon atrophic course associated with delayed presentation and persistent MNV activity. These two examples were chosen to illustrate the spectrum of outcomes observed in the cohort.

### Safety Outcomes

Safety outcomes were favourable across the cohort. One patient developed transient intraocular pressure elevation (Case 9), which resolved following anterior-chamber paracentesis. Two patients experienced non-vision-threatening cataract progression during follow-up (Cases 7 and 8), while two eyes showed MNVB scarring (Cases 3 and 10). Importantly, no episodes of endophthalmitis, retinal detachment, retinal pigment epithelium tear, recurrent or breakthrough haemorrhage were observed throughout the six-month follow-up period.

## 4. Discussion

Sub-macular haemorrhage in neovascular AMD progresses quickly and can cause permanent visual loss. Blood trapped under the retina separates the photoreceptors and exposes them to iron and fibrin, which accelerates damage. These mechanisms are well described, and they explain why prompt treatment is generally associated with better outcomes [4,5]. In our series, BCVA improved significantly over time based on repeated-measures analysis, with most eyes showing recovery of foveal contour on OCT. This was consistent with recently published analysis, which revealed that combining tPA and anti-VEGF resulted in significant visual acuity improvement (−0.38 logMAR at 1 month, −0.47 logMAR at longer follow-up) with an 86% success rate in haemorrhage displacement [8].

The two eyes in our series that developed atrophy had the longest delay to treatment start. It supports the broader view that timing may be more important than the specific treatment technique [19]. Cases 3 and 10 showed limited visual recovery and developed MNV-related scarring. Both eyes presented late, with haemorrhage duration up to 2 months, and OCT demonstrated early ellipsoid-zone disruption. Prolonged contact of the retina with iron-rich blood likely accelerated photoreceptor apoptosis, while persistent MNV activity, visible on OCT-A, promoted fibrovascular proliferation [4,5]. These mechanisms are consistent with prior studies showing that delayed treatment and active neovascular complexes significantly increase the risk of sub-foveal scarring [20,21]. Moreover, it was also emphasised that the increased risk of breakthrough and other complications occurs when triple therapy is delayed [22].

The clear gap between the mean and median duration of symptoms (Table 1) reflects a skewed distribution. Three patients presented after more than 50 days, which increased the mean markedly. The median of 12 days better reflects the typical presentation in this cohort. Such delays are common in elderly patients who may misinterpret symptoms or postpone care. These outliers also help explain differences in anatomical outcomes across the group [19,21].

Our protocol combined intravitreal alteplase with pure C_3_F_8_ gas, followed by aflibercept within a treat-and-extend regimen. Both the 2 mg and 8 mg aflibercept formulations were pooled for analysis because they operate through the same mechanism and are used interchangeably in clinical practice [18]. The purpose of this study was not to compare the two formulations but to document outcomes using a consistent real-world treatment pathway. Although the 8 mg dose may allow faster extension to 12-week intervals, the short six-month window of this study does not allow meaningful differentiation between the two dosing strategies [18]. When compared with published reports, our results fall within the expected range for minimally invasive displacement techniques. Several studies have shown that pneumatic displacement with or without intravitreal tPA can achieve substantial anatomical clearance and meaningful visual improvement [9,10]. Recent evidence also suggests that less invasive protocols may offer results like vitrectomy-based approaches in selected cases [8]. Similarly, combining low-dose subretinal tPA with intravitreal anti-VEGF during vitrectomy produced favourable six-month outcomes, although retinal pigment epithelium (RPE) degeneration in some patients highlights the need for individualised treatment planning [3,9,22].

Notably, a recent randomised trial indicated that surgical management of SMH—vitrectomy with subretinal tPA and intravitreal gas—did not significantly outperform pneumatic displacement in improving visual outcomes [3,10]. The ongoing TIGER trial is expected to provide clarity on surgical versus non-surgical strategies in large, fovea-involving haemorrhages [7,23]. Announced in 2020 and updated in 2025, this trial is still ongoing, with no results published yet [15,23].

The present study adds current, OCT-documented, short-term outcomes using a protocol that is both accessible and feasible in outpatient settings. Recent advances in anti-angiogenic research also broaden the context of our findings. Experimental work using nanotechnology-based delivery systems has shown promising modulation of pathological angiogenesis in neovascular AMD, suggesting new directions for future therapeutic refinement [24]. Including such innovations helps balance current clinical outcomes with emerging translational perspectives.

The study has clear limitations. The sample is small, there is no control group, and the retrospective design limits causal interpretation. Although early-treated eyes showed a tendency toward greater visual gains, the sample is too small for formal subgroup inference. The follow-up is limited to six months, and longer observation would be needed to document recurrence, long-term stability of the outer retina, and differences between aflibercept formulations.

Despite these limitations, the consistent direction of both visual and anatomical improvement supports the potential value of this minimally invasive combined approach. Early intervention appears to be an important factor [10,19]. Larger prospective studies with defined comparison groups are needed to clarify efficacy, identify ideal timing, and refine anti-VEGF dosing strategies.

## 5. Conclusions

This case series indicates that combined intravitreal alteplase and pure C_3_F_8_ gas, followed by anti-VEGF therapy, may offer favourable short-term visual and anatomical improvement in SMH secondary to nAMD. Early treatment appears important. Larger prospective studies are required to validate these findings and optimise patient selection and dosage protocols.

## Figures and Tables

**Table 1:** Demographic data with diagnosis and the onset of symptoms. F = female, M = male, AMD-age-related macular degeneration.

Case No.	Age (Years)	Sex	Initial Diagnosis	Duration of Symptoms (Days)
1	90	F	Sub-macular haemorrhage, AMD	60
2	85	F	Sub-macular haemorrhage, AMD	7
3	74	M	Sub-macular haemorrhage, AMD	21
4	78	M	Sub-macular haemorrhage, AMD	10
5	78	M	Sub-macular haemorrhage, AMD	7
6	68	M	Sub-macular haemorrhage, AMD	14
7	73	M	Sub-macular haemorrhage, AMD	7
8	77	F	Sub-macular haemorrhage, AMD	56
9	60	F	Sub-macular haemorrhage, AMD	60
10	77	F	Sub-macular haemorrhage, AMD	8
**Mean**	**76**		**—**	**24**
**Median**	**75**	**—**	**—**	**12**

**Table 2:** Best-corrected visual acuity (BCVA) change with time (in logMAR).

Case No.	BCVA Before	7–14 Days Postop.	1 Month Postop.	3 Months Postop.	6 Months Postop.
1	1.00	0.70	1.00	0.52	0.52
2	1.30	0.70	0.70	0.52	0.40
3	2.00	2.00	2.00	1.40	1.40
4	1.30	1.30	0.52	0.40	0.30
5	0.30	0.52	0.52	0.40	0.40
6	0.60	0.60	0.60	0.52	0.22
7	0.40	0.40	0.52	0.52	0.52
8	1.00	1.00	0.70	0.52	0.52
9	0.70	0.40	0.30	0.22	0.10
10	1.30	1.30	1.10	1.10	1.10
**Mean**	**0.99**	**0.89**	**0.80**	**0.60**	**0.53**
**Median**	**1.00**	**0.70**	**0.65**	**0.52**	**0.40**

## Data Availability

The data presented in this study are available on request from the corresponding author due to Polish law protecting patient’s data -privacy and ethical restrictions.
